# A Guild of 45 CRISPR-Associated (Cas) Protein Families and Multiple CRISPR/Cas Subtypes Exist in Prokaryotic Genomes

**DOI:** 10.1371/journal.pcbi.0010060

**Published:** 2005-11-11

**Authors:** Daniel H Haft, Jeremy Selengut, Emmanuel F Mongodin, Karen E Nelson

**Affiliations:** The Institute for Genomic Research, Rockville, Maryland, United States of America; The Institute for Genomic Research, United States of America

## Abstract

Clustered regularly interspaced short palindromic repeats (CRISPRs) are a family of DNA direct repeats found in many prokaryotic genomes. Repeats of 21–37 bp typically show weak dyad symmetry and are separated by regularly sized, nonrepetitive spacer sequences. Four CRISPR-associated (Cas) protein families, designated Cas1 to Cas4, are strictly associated with CRISPR elements and always occur near a repeat cluster. Some spacers originate from mobile genetic elements and are thought to confer “immunity” against the elements that harbor these sequences. In the present study, we have systematically investigated uncharacterized proteins encoded in the vicinity of these CRISPRs and found many additional protein families that are strictly associated with CRISPR loci across multiple prokaryotic species. Multiple sequence alignments and hidden Markov models have been built for 45 Cas protein families. These models identify family members with high sensitivity and selectivity and classify key regulators of development, DevR and DevS, in *Myxococcus xanthus* as Cas proteins. These identifications show that CRISPR/*cas* gene regions can be quite large, with up to 20 different, tandem-arranged *cas* genes next to a repeat cluster or filling the region between two repeat clusters. Distinctive subsets of the collection of Cas proteins recur in phylogenetically distant species and correlate with characteristic repeat periodicity. The analyses presented here support initial proposals of mobility of these units, along with the likelihood that loci of different subtypes interact with one another as well as with host cell defensive, replicative, and regulatory systems. It is evident from this analysis that CRISPR/*cas* loci are larger, more complex, and more heterogeneous than previously appreciated.

## Synopsis

The family of clustered regularly interspaced short palindromic repeats (CRISPRs) describes a class of DNA repeats found in nearly half of all bacterial and archaeal genomes. These DNA repeat regions have a remarkably regular structure: unique sequences of constant size, called spacers, sit between each pair of repeats. The DNA repeats do not encode proteins, but appear to be transcribed and processed into small RNAs that may have any number of functions, including resistance to any phage (i.e., virus of bacteria) whose sequence matches a spacer; spacers change rapidly as microbial strains evolve. This work describes 41 new CRISPR-associated *(cas)* gene families, which are always found near these repeats, in addition to the four previously known. It shows that CRISPR systems belong to different classes, with different repeat patterns, sets of genes, and species ranges. Most of these seem to come and go rather rapidly from their host genomes. These possibly beneficial mobile genetic elements may play an important role in driving prokaryotic evolution.

## Introduction

Clusters of short DNA repeats with nonhomologous spacers, which are found at regular intervals in the genomes of phylogenetically distinct prokaryotic species, comprise a family with recognizable features [1–3]. These repeats were first observed by Ishino and colleagues [4] upstream of the *iap* gene in *Escherichia coli* and later in other bacterial and archaeal species such as *Haloferax mediterranei, Streptococcus pyogenes,* and *Mycobacterium tuberculosis.* Each member of this family of repeats was designated differently by the original authors, leading to a confusing nomenclature: SPIDR (spacers interspaced direct repeats) by Jansen and colleagues [5], SRSR (short regularly spaced repeats) by Mojica and colleagues [2], and LCTR (large cluster of 20-nt tandem repeat sequences) by She and colleagues [6], among others. Based on a systematic characterization in 40 different bacterial and archaeal genomes, Jansen and colleagues [3] proposed, in agreement with Mojica's research group, a new nomenclature for this family of DNA repeats, which are now known as clustered regularly interspaced short palindromic repeats (CRISPRs). Sequencing of the genome of the archaeon *Methanococcus* (now *Methanocaldococcus*) *jannaschii* [7] led to the first detailed characterization of these repeats in a complete genome, where 18 loci were found, most featuring a single copy of a long repeat (LR) or leader sequence and a variable number of regularly spaced short repeats (SRs). In *M. jannaschii,* two of the LRs were truncated, each ending with a single SR, and one cluster of five SRs had no LR. Similar repeats were identified in the genome of the hyperthermophilic bacterium *Thermotoga maritima* [8]. The association of these repeats with nearby gene clusters that showed closest similarity to archaeal species and their atypical DNA composition (as measured by χ^2^ analysis) were called consistent with other observations as evidence of lateral gene transfer (LGT) between archaeal and bacterial species [8]. Together, these findings suggested transfer of repeat-associated DNA within and between prokaryotic genomes.

Four genes, designated CRISPR-associated *(cas),* are found only in species containing CRISPR, always located near to a repeat locus, and usually oriented head-to-tail as if cotranscribed [3]. The most common arrangement of these genes is *cas3*–*cas4*–*cas1*–*cas2.* The Cas3 protein appears to be a helicase, whereas Cas4 resembles the RecB family of exonucleases and contains a cysteine-rich motif, suggestive of DNA binding. Cas1 is generally highly basic and is the only Cas protein found consistently in all species that contain CRISPR loci. Cas2 remains to be characterized. None of the other genes in the vicinity of these four *cas* genes were found to represent protein families specifically associated with CRISPR.

There has been only limited biological characterization of these elements. Efforts to increase the copy numbers of these repeats in the archaeon *Haloferax volcanii* resulted in altered segregation and reduced viability of the cells [1]; a role in replicon partitioning was suggested. Supporting this, small clusters of repeats are found in two self-transmissible plasmids of the genus *Sulfolobus;* these plasmids appear more stably maintained than plasmids lacking repeats [9]. The main chromosome of *Sulfolobus solfataricus,* unlike the plasmids themselves, has both *cas* gene clusters next to repeats [3] and a genus-specific binding protein (SSO0454) for its own and for the plasmids' repeats [10]. Tang and coworkers [11] found 22 small, nonmessenger RNAs transcribed from CRISPR arrays of the archaeon *Archaeoglobus fulgidus,* nearly all had sizes just below a repeat plus a spacer with the 3′ end in the middle of the repeat; repeat arrays of *S. solfataricus* also were transcribed into RNA and processed. Two recent analyses of spacer elements found between individual CRISPRs demonstrate that most have no conclusive origin by sequence similarity; those that do, strikingly, match phage or other types of selfish genetic elements [12,13]. Despite these advances, the functions of both CRISPR and Cas proteins remain unknown. In this study, we present a detailed analysis of four previously defined and 41 newly identified Cas proteins in the microbial species for which we have complete genome sequences.

## Results

### Identification of New CRISPR-Associated (Cas) Protein Families

In addition to the previously described *cas* genes *(cas1–4),* we have detected a number of protein families whose members are found in the vicinity of CRISPRs across multiple prokaryotic species. Hidden Markov models (HMMs) for these families have been constructed and deposited in the TIGRFAMs database (http://www.tigr.org/TIGRFAMs; Table 1). In the present work, the CRISPR-associated protein families are described as a “guild,” i.e., a collection of members that perform somewhat similar work. The guild is presumed to be involved in processes that may include the maintenance of repeat clusters [3], capture of new spacer elements [12,13] and expansion or contraction of clusters, propagation of the leader sequence and repeat clusters within a genome [3,7], transfer of CRISPR and *cas* genes together to new genomes [8,14,15], and interaction of CRISPR/*cas* loci with the host cell (see Discussion). From our study, a total of 53 HMMs have been constructed that represent at least 45 different protein families (including Cas1–4). The discrepancy between the number of HMMs and protein families results from two pairs of models for the Cas2 protein and the Cas3 protein (Table 1), which have enough sequence divergence that a single model is not sufficient. Also included is a model for the HD domain of Cas3, which in some cases is a separate polypeptide and in others is absent. Finally, in addition to a model that detects the diagnostic domain of Cas5 (see below), we present five narrower models that detect the five subtype-specific full-length variants of this family (Table 1). Many of these families contain members that belong to clusters of orthologous groups (COGs) [14,16], although the relationship between the HMMs described here and these COGs is imperfect (see Discussion). The functions of these protein families are largely unknown, although distant homologies to characterized proteins, motifs, and domains have been noted in the present study and in previous analyses [14] (Table 1). For example, eight families of CRISPR-associated proteins (Csm3–5, Cmr1, Cmr3, Cmr4, Cmr6, and Csx7) all belong to the repair-associated mysterious protein (RAMP) superfamily [14] as detected by Pfam [17] model PF03787. These RAMP families appear to act in concert since sets of them typically are found in gene clusters (Figure 1).

**Table 1:**
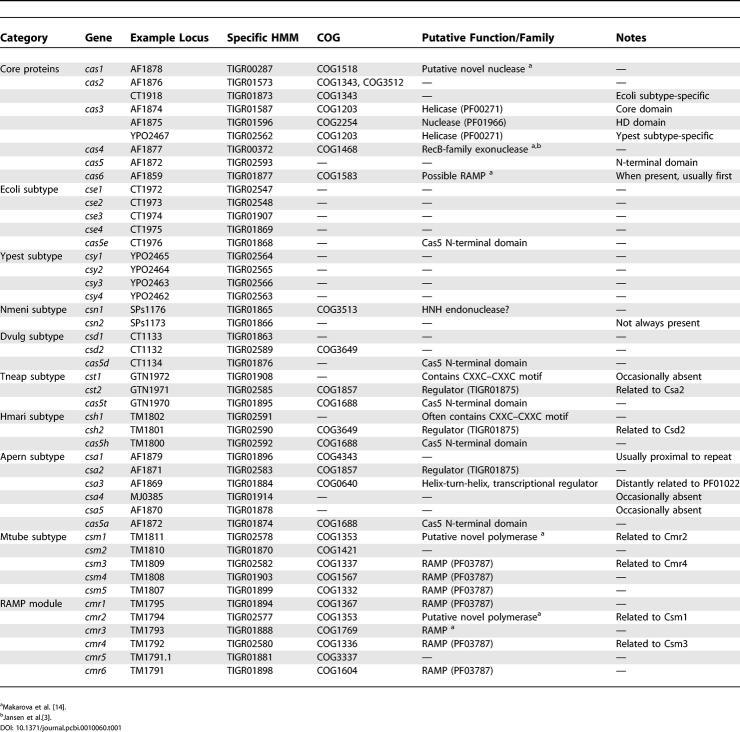
Description of the Different *cas* Core Genes, CRISPR/Cas Subtypes, and the RAMP Module, Based on the New Cas Protein Families

**Figure 1:**
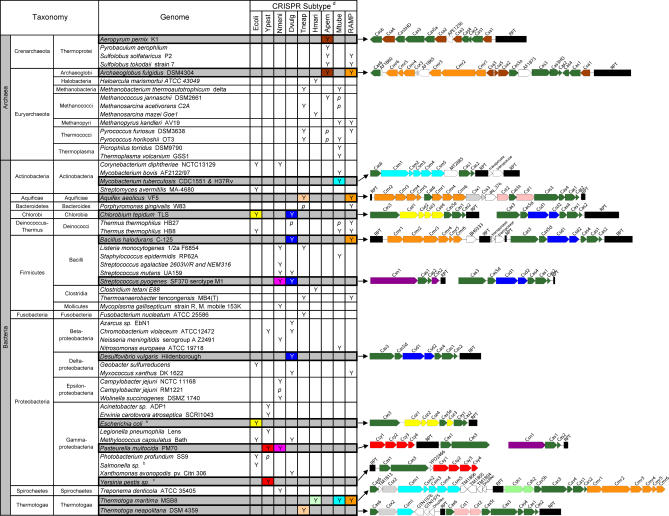
Distribution of the Different CRISPR/Cas Subtypes across Some of the Prokaryotic Species for Which a Whole-Genome Sequence Is Available The taxonomy of each species/strain is indicated on the left side of the figure. The CRISPR/*cas* loci of a number of illustrative examples for the different CRISPR subtypes are displayed on the right side of the figure. ^a^ *E. coli* K12-MG1655, O157:H7 EDL933, and O157:H7 VT2-Sakai. ^b^ *Salmonella enterica* Paratyphi ATCC9150, serovar Typhi CT18, and Ty2; *Salmonella typhimurium* LT2 SGSC1412. ^c^ *Y. pestis* CO92, KIM, and biovar Mediaevalis 91001; *Yersinia pseudotuberculosis* IP32593. ^d^“*p*” indicates a partial cluster lacking some of the genes usually associated with this subtype, the repeats, or both. Such clusters may represent autonomous functional units, degradation from the common subtype, or cases in which the missing components are supplied by distantly located CRISPR clusters within the same genome.

The assignment of genes to these 45 families has allowed for an analysis of the genomic context in which they are typically found. Three basic types of family context have emerged. First, the “core” *cas* genes (i.e., *cas1–4*) are found in a wide range of contexts with respect to the other gene families, whose genes are clustered nearby. Second, subtype-specific genes are found in association with the core genes and others of the same subtype, often with conserved gene order. Finally, modular genes, associated with one another in particular combinations, are always found in genomes containing the core genes, but may be found at distant sites from those clusters.

Based on the observation of such contextual patterns, we have defined two additional core *cas* genes (*cas5* and *cas6*), eight CRISPR-associated subtypes, and one CRISPR-associated module each of which is described in detail below and presented in Table 1. Each of the subtypes has been named for a genome in which it appears as the only CRISPR locus (e.g., CRISPR subtype Apern after *Aeropyrum pernix;* see Table 1, Figure 1), and the associated subtype-specific genes have been assigned gene symbols based on a three-letter prefix and a numeral suffix (i.e., *csa1*) following the *cas1–4* model (Table 1, Figure 1). The module (CRISPR RAMP module, *cmr1–6*) has been named after the RAMP superfamily since four of the six genes appear to be members of this superfamily. Models for a number of families have been constructed for which no contextual pattern has yet been defined, most likely due to an insufficient number of genomes harboring the gene. These have been assigned gene symbols with the prefix *“csx”* (Table 2).

**Table 2:**
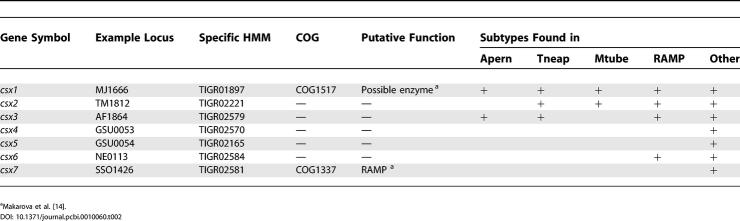
Other CRISPR/Cas Protein Families with No Identified Contextual Pattern

Our assignments of genes to CRISPR-associated families has allowed for the identification of CRISPR/cas loci that span the genomic distance between CRISPR arrays not previously appreciated as forming the same locus (e.g., *Bacillus halodurans* C-125 and *Aquifex aeolicus* VF5; see Figure 1). Indeed, it appears to be a rule that virtually every gene found between any two *cas* genes is strictly CRISPR-associated itself, although it may not be as common as the core *cas* genes, *cas1–cas6.* The exceptions are putative transposases, restriction enzymes, and addiction module proteins, or hypothetical proteins with few or no homologs; several examples appear in Figure 1. Frequently we have found that the addition of new genomes to our databases shows such hypothetical proteins to belong to a new family of *cas* genes. Through this process of slowly building up our library of *cas* gene families, the patterns of conserved subtypes, previously obscure, has come into sharp focus (Figure 1).

### New Core *cas* Gene Families: *cas5* and *cas6*

The *cas* core genes *(cas1–4)* were originally delineated by Jansen and colleagues [3] and are characterized by their close proximity to the CRISPR loci and their broad distribution across bacterial and archaeal species. Although not all *cas* core genes associate with all CRISPR loci, they are all found in multiple subtypes (Table 3). We have observed a 43-amino acid N-terminal domain, which appears in a single protein in each of five separate CRISPR/Cas subtypes and in a number of currently untyped loci. We designate these families collectively as Cas5. Members average 250 amino acids in length; regions outside the N-terminal domain form subtype-specific families with remote to undetectable homology across subtypes. For this reason, we have included both subtype-specific full-length models and the domain model in the TIGRFAMs library (see Table 1) and have assigned gene symbols with a trailing letter to indicate the subtype variant in question (e.g., *cas5e,* for the Ecoli subtype variant). The Cas5 domain is found in the *M. xanthus* DK1622 DevS protein, which has been implicated in a species-specific developmental pathway [18,19], although found within an apparent CRISPR/*cas* locus of a novel subtype. There may be a distant homology relationship between Cas5 proteins and the RAMP superfamily, though not detected by the Pfam [17] RAMP superfamily model PF03787 (data not shown).

**Table 3:**
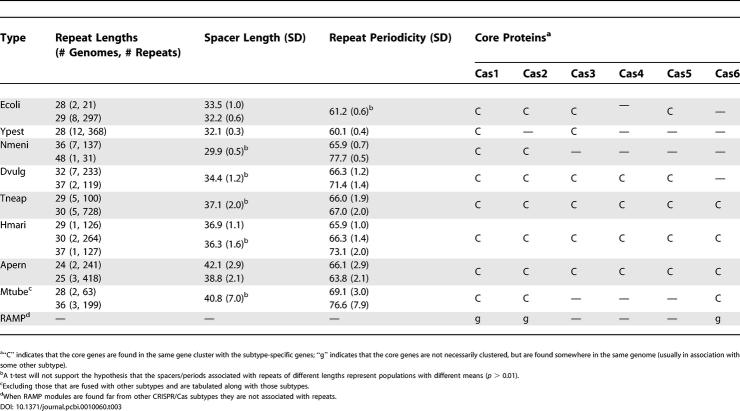
Characteristics of the Repeat Arrays Associated with the Different CRISPR/Cas Subtypes

The Cas6 family includes proteins averaging 140 amino acids in length that share a strong homology at the C-terminus, including a GhGxxxxxGhG motif (“h” indicates a hydrophobic amino acid). The *cas6* gene is found in association with four separate CRISPR/Cas subtypes (see Table 3) and has a preferred location as the *cas* gene most distal to the CRISPR (Figure 1).

### Description of the Different CRISPR/Cas Subtypes

Tables 1, 3, and 4 and Figure 1 delineate the essential features of the eight CRISPR/Cas subtypes that we have defined thus far, including the subtype-specific (see Table 1) and core (see Table 3) genes involved, the length and periodicity of the repeats and the length distributions of the associated spacers (Table 3), the co-occurrences and subtype fusions observed (see Table 4), and the species in which they are found (Figure 1). Distinctive and notable features of these subtypes will be discussed below. Each subtype is named for the species of a genome sequence in which only that subtype is found.

**Table 4:**
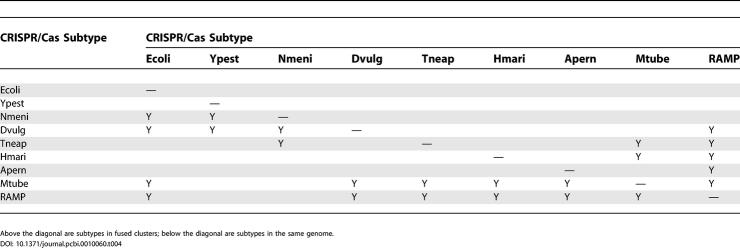
Co-Incidence of the Different CRISPR/Cas Subtypes

### CRISPR/Cas Subtype Ecoli (Based on *Escherichia coli* K12-MG1655)

The Ecoli subtype features five subtype-specific genes and *cas1–3.* The *cas2* gene associated with this subtype is sufficiently diverged from the rest of the Cas2 family so that the construction of a separate HMM (TIGR01873) was necessary. The 61-bp average (see Table 3) periodicity is unique among the subtypes we describe, and we never find an Ecoli-type cluster fused to another type. This subtype is sporadically distributed among bacteria and not found in any of the sequenced Archaea present in the Comprehensive Microbial Resource (http://www.tigr.org/tigr-scripts/CMR2/CMRHomePage.spl) [20]. Saunders and coworkers [21] report a cluster of “bacterial-specific” CRISPR-associated genes in the incomplete genomic sequence of the cold-adapted archaeon *Methanococcoides burtonii* that prove to be *cas3, cse1, cse2,* and *cse4;* we detect a second cluster in this organism with the remaining four required genes.

### CRISPR/Cas Subtype Ypest (Based on Various *Yersinia pestis* Strains)

The Ypest subtype is unique in its lack of a Cas2 homolog. It has the shortest average repeat periodicity (only 60 bp; Table 3), the most well-conserved repeat sequence from one species to another, and easily the narrowest phylogenetic range. It is observed only in several *Gammaproteobacteria* sp. and one *Betaproteobacterium.* The Cas3 putative helicase associated with this subtype is sufficiently diverged from the rest of the Cas3 family that the construction of a separate HMM (TIGR02562) was necessary. It is the spacers of the Ypest-subtype repeats in *Y. pestis,* which were analyzed by Pourcel and colleagues [13]. The *cas1* gene associated with these clusters is most closely related to that of the Ecoli subtype (Figure 2), which has the next shortest repeat periodicity.

**Figure 2:**
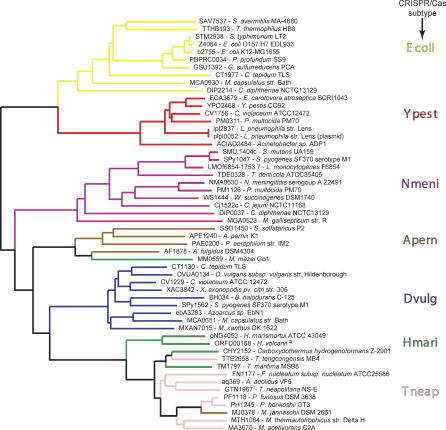
Molecular Phylogeny of the Cas1 Protein across 54 Prokaryotic Genomes A representative selection of Cas1 protein sequences were aligned using ClustalW, and columns with greater than 20% gaps were removed. A neighbor-joining tree was calculated in Belvu using the Storm and Sonnhammer distance correction. Trees calculated using more computationally intensive methods showed insignificant differences. ^a^From the preliminary annotation of the *Haloferax volcanii* genome, currently sequenced at The Institute for Genomic Research (http://www.tigr.org/tdb/mdb/mdbinprogress.html).

### CRISPR/Cas Subtype Nmeni (Based on *Neisseria meningitidis* Serogroup A Z2491)

This subtype is the most abbreviated that we have described, being the only one lacking *cas3, cas4,* and *cas5* and having the shortest average spacer lengths observed (30 bp; Table 3). Jansen and colleagues [3] noted similarity between Cas4 and RecB family exonucleases. Members of the Csn1 family, by contrast, contain an McrA nuclease-like domain [14]. Csn1, a large and likely multidomain protein, may perform the functions of the absent Cas4 and potentially Cas3 as well. A second subtype-specific gene, *csn2,* is present in some but not all Nmeni CRISPR/*cas* loci. A characteristic feature of this subtype is a single copy of the repeat (sometimes direct, sometimes inverted), which appears upstream of the first gene in the locus, in addition to the repeat cluster downstream of the last gene. Notably, species bearing this CRISPR/Cas subtype are, without exception, vertebrate pathogens and commensals.

### “Three-Gene” CRISPR/Cas Subtypes: Dvulg (Based on *Desulfovibrio vulgaris* Hildenborough), Tneap (Based on *Thermotoga neapolitana* DSM4359), and Hmari (Based on *Haloarcula marismortui* ATCC 43049)

A number of subtypes appear to have a similar overall structure consisting of *cas1–4* (and, with the exception of Dvulg [see below] of *cas6* as well) and three subtype-specific genes, including the subtype-specific *cas5* variants (see Figure 1). Typically, one of these shows homology to the DevR protein, which has been characterized as a negative autoregulator and is the presumed partner of DevS/Cas5 from *M. xanthus* DK1622 [22], while the last is a large (400–700 amino acids) protein, often containing a CXXC–CXXC motif. These three genes are always adjacent to *cas3.* Each of these types generally is associated with repeat spacers of a distinct average length (Dvulg, 34; Hmari, 36; and Tneap, 37; Table 3), and all have *cas1* genes that are more closely related to one another than to the *cas1* genes of other subtypes (see Figure 2). Several genomes contain CRISPR/*cas* loci that also appear to conform to this structural class, but the number of sequenced genomes containing homologs is currently insufficient to create subtype-specific HMMs (e.g., CRISPR/*cas* loci of *Leptospira interrogans* serovar Lai strain 56601 and *Fusobacterium nucleatum* ATCC 25586). In addition to the repeat cluster immediately downstream of the *cas* gene operon, CRISPR/*cas* loci of the Tneap and Hmari types, but not the Dvulg type, frequently have another cluster immediately upstream.

### CRISPR/Cas Subtype Apern (Based on *A. pernix* K1)

This subtype is found only in Archaea and comprises the only described subtype in the Crenarchaeota (although the RAMP module is also observed; see below). Although this subtype is only found in thermophilic species, significance of this correlation is tempered by the fact that the large majority of archaeal species are thermophiles. In *Sulfolobus* sp. and *A. fulgidus* DSM4304, *csa4* is absent, while *csa5,* a gene not found in *A. pernix,* is present, although they have no detectable homology. In similar fashion to the three-gene class of subtypes (see above), *csa2* is a distant homolog of *devR.* The *cas1* genes of this subtype are most closely related to those of the three-gene subtypes; indeed, the *cas1* gene of *M. jannaschii* DSM2661 would appear by homology to be a Tneap type and may represent an instance of subtype recombination (Figure 2).

### CRISPR/Cas Subtype Mtube (Based on *M. tuberculosis* Strains CDC1551 and H37Rv)

Although observed as the sole subtype in several genomes, this subtype is more commonly found in genomes containing other subtypes at remote sites and in hybrid, fused loci (e.g., *T. maritima;* see Figure 1). The subtypes with which Mtube have been observed to associate are Tneap, Hmari, and Apern (Figure 1). The repeats proximal to the Mtube genes in unfused loci have long, but variable average periodicities and spacers (Figure 3). When found in fused loci, the *csm* genes tend to be distal from *cas1* and *cas2,* which are themselves proximal to the subtype-specific genes of the other subtype in the locus. Additionally, in these hybrid loci, the spacer length is typical of the subtype partner, not of the Mtube subtype. Occasionally, as is observed in *Methanosarcina acetivorans* C2A, an Mtube locus with a robust repeat array is found lacking all *cas* core genes, although they are found elsewhere in the genome in association with a CRISPR/*cas* locus of another subtype. This would suggest that the linkage between the core *cas* genes and the subtype-specific genes is weaker in this subtype and is somewhat akin to the behavior of the RAMP module (see below). The notable variability of the spacer lengths may also indicate a heterogeneity of origin for these repeat arrays. When the *cas1* genes that are proximate to *csm* genes are incorporated into a tree such as the one displayed in Figure 2, they do not form a single clade, but are found in clades that include *cas1* genes associated with those subtypes with which Mtube typically associates (data not shown). Additionally, three of the five subtype-specific genes *(csm3–5)* are members of the RAMP superfamily, and *csm1* is a homolog of *cmr2.*

### CRISPR/Cas RAMP Module

CRISPR/Cas systems include a six-gene module that appears to occur only in genomes that contain other CRISPR systems, whether or not those systems are nearby. The cluster of genes *cmr1–6* is observed in a wide range of archaeal and bacterial genomes, but always in the same genome with other subtypes, and most often fused into hybrid loci (see Figure 1; Table 1). Four of the six genes in this module are members of the RAMP superfamily [14]. This RAMP module associates with the three-gene class (Dvulg, Tneap, and Hmari), as well as Apern and Mtube subtypes. We have observed one instance where a RAMP module is found in the same genome with an Ecoli type (in *Thermus thermophilus* HB8), but in this case the RAMP is actually part of a hybrid locus with an Mtube subtype.

### Degraded and Atypical CRISPR/*cas* Loci

Expansion of the number of CRISPR families from four to 45, definition of CRISPR/Cas subtypes, and examination of over 200 genomes allow reexamination of *cas* pseudogenes and degenerate CRISPR/*cas* loci. The genome of *Thermoplasma volcanium,* e.g., should be viewed not as having lost much of the system present in many other Archaea [14], but rather as having a CRISPR/*cas* locus of the Mtube type, while many other Archaea have loci of the Apern type (Figure 1). We believe we have observed evidence of ongoing processes of CRISPR locus degradation in multiple independent cases in five separate subtypes (Ecoli, Ypest, Nmeni, Dvulg, and Mtube) in bacteria. Examples include both *cas1* and *cas2* pseudogenes adjacent to apparently intact subtype-specific genes (allowing subtype identification) and loci in which novel subtype-specific genes are degenerate. A dramatic case of degradation is found in the genome of *Coxiella burnetii* RSA 493, in which the Ypest locus contains frameshifts and truncations in four of the six genes, and the repeat array consists of only a single exact copy of the Ypest repeat. The Ypest repeat is so well conserved that it can be used to search for genomes in which it is the only remaining trace of a Ypest CRISPR locus. BLAST searches of a consensus sequence created from alignments of the repeat can detect instances with up to four mismatches. We have detected tiny arrays of one or two full-length copies and one additional partial repeat (with the expected spacing of exactly 60 nt) only in several strains of *E. coli, Shigella flexneri,* and *Shewanella oneidensis* (all of which are within the phylogenetic range of the Ypest subtype generally).

Atypical CRISPR systems do occur, such as the previously overlooked repeat array in the genome of *Thermoplasma acidophilum,* where no *cas* genes are found, and the Ypest system of *Zymomonas mobilis* ZM4, where the *cas* gene cluster is far from the characteristic 28-bp repeat cluster. Large distances between *cas* gene clusters and their closest repeat clusters occur less often than *cas* pseudogenes adjacent to degenerate repeats. Excluding these rare exceptions, the average distance from the *cas* gene cluster to the nearest repeat cluster is well below 1,000 bp and varies according to CRISPR/Cas subtype, e.g., 180 bp for Dvulg, 232 bp for Ypest, and 414 bp for Apern. This spacing often accommodates the CRISPR leader sequence.

### Molecular Phylogeny of Cas Core Proteins

The definition of the subtypes discussed above was driven by the observation of the conserved contexts of families of distinct genes. As has been mentioned above, the *cas* core genes are found across various subtypes, most definitively in the case of *cas1,* which appears to be nearly universal for CRISPR/*cas* loci. The molecular phylogeny of various Cas core proteins has been explored by the construction of multiple sequence alignments, restriction of those alignments to well-aligned regions, and the calculation of neighbor-joining trees. A representative tree for Cas1 is shown in Figure 2. Trees for other Cas core proteins showed largely the same pattern, although limited to the subtypes in which they are individually found. These trees were robust, showing insignificant differences in branching patterns when a variety of alignment regions and tree-building algorithms were used. The clustering of the Cas core proteins broadly recapitulates the subtype divisions that were defined independently of this information. There would appear to be a limited number of cases where the Cas core proteins do not share the same evolutionary history as their associated subtype-specific proteins.

## Discussion

CRISPR is a widely distributed family of repeats in prokaryotes [1–3,5,7,15]. Preliminary insight into their biology came with the discovery that four different protein families occur in prokaryotes only if CRISPRs are present. These proteins are always near a set of these repeats and always include Cas1 [3]. In the current study, we built on these prior findings and established a number of HMM-defined Cas protein families. These protein families have been found to form conserved clusters across multiple genomes, which allowed us to create rules for the identification of specific subtypes of CRISPR/Cas system.

From the study presented here, it is apparent that CRISPR/Cas systems are far more complex than previously appreciated. Forty-five distinct protein families associated with CRISPRs have been identified among the first 200 completed prokaryotic genomes. These are currently represented by 53 HMMs (Tables 1 and 2). These models are sensitive, in that they unambiguously identify genes, and are also selective, in that they do not identify genes in organisms lacking CRISPR/*cas* loci. The subtype-specific models accurately discriminate between the subtypes but may, infrequently, identify genes in novel CRISPR/*cas* contexts that, given sufficient additional genomes, would warrant the status of separate subtypes.

Previous work by Makarova and colleagues [14], conducted on a smaller set of available microbial genomes and without the knowledge of the associated CRISPRs, resulted in the identification of some 20 gene families (COGs) proposed to act in DNA repair, many of which contain genes identified by our HMM models. The relationship between these two sets of families is uneven, with some of our HMMs spanning multiple COGs, some COGs spanning multiple HMMs, and some COGs including genes we believe unrelated to CRISPRs. COG0640, e.g., includes eight putative transcriptional regulators in *A. fulgidus* and five in *M. jannaschii,* but only MJ0379 and AF1869, one locus in each species, are CRISPR-associated; they encode the Csa3 protein of the Apern-type CRISPR system. These differences are not unexpected, considering the different clustering methods and search algorithms applied to unequal datasets in this case. Their work also introduced the RAMP superfamily[14], to which a number of Cas protein families belong. The proposed helicase, nuclease, and other domains for DNA repair metabolism may instead or in addition act in the processes of CRISPR physiology: mobilization, maintenance, processing, and addition of new spacer elements. To reflect this change in interpretation, we propose renaming the RAMP superfamily from repair-associated to repeat-associated mysterious protein, thus preserving the acronym currently in use.

The groups of gene families that comprise the CRISPR/Cas subtypes appear to have traveled together through evolutionary time as discrete units. Even the core *cas* genes appear to have the same evolutionary history as their partner subtype-specific genes (Figure 2). The reasonable hypothesis that the Cas proteins interact (i.e., bind to, stabilize, regulate the expression of, cleave, modify, degrade, etc.) with the repeats in their DNA or expressed RNA form is supported by the observation of subtype-specific characteristics of the repeats such as repeat periodicity. Although as demonstrated in this study, CRISPR/*cas* loci of different subtypes can coexist within the same genome, phylogenetic reconstructions of Cas core proteins do not provide any evidence of switching between subtypes having repeat periodicities of 60, 61, and those with longer periodicities (Figure 2). The RAMP module and the RAMP-like Mtube subtype would appear to deviate from this pattern, showing varying degrees of independence from dedicated *cas* core genes and their associated repeat periodicities.

It has been previously suggested that *cas* genes have undergone LGT events based on phylogenetic analyses and conservation of gene order [14], anomalous nucleotide frequencies [8], and the presence of multiple chromosomal CRISPR loci [3]. Our finding that the core *cas* genes belong to multiple CRISPR subtypes, each with its own sporadic distribution, indicates that this conclusion should be reexamined and reconfirmed. Indeed we have observed several lines of evidence that support the LGT hypothesis: (1) CRISPR/*cas* loci representing five different subtypes are found on plasmids (subtype Ypest in *Legionella pneumophila* Lens, subtype Dvulg in *D. vulgaris,* subtype Hmari in *H. marismortui,* subtype Ecoli in *Photobacterium profundum,* and both subtypes Mtube and Ecoli in *T. thermophilus* HB8). In the case of *L. pneumophila* Lens, a second, nearly identical copy of the locus is found on the chromosome. (2) In *L. pneumophila* Paris, by contrast, there is no trace of any gene with homology to the Ypest subtype genes or repeats found in the Lens strain, while an entirely different (untyped) CRISPR locus is found. Differences in CRISPR locus content have been observed between closely related strains of *S. pyogenes, Listeria monocytogenes,* and *T. thermophilus.* (3) Comparison of the Ecoli subtype loci from *E. coli* K12-MG1655 and *E. coli* O157:H7 EDL933 shows that while Cas1, Cas2, and the surrounding genomic region are nearly identical between K12-MG1655 and O157:H7 EDL933, this similarity does not extend to the rest of the Cas proteins in the cluster. For K12-MG1655, these proteins are most similar to those in *Geobacter sulfurreducens,* while for O157:H7 EDL933 they are most similar to those of *Photorhabdus luminescens.* (4) Cas1 proteins found in *Porphyromonas gingivalis* W83, *Vibrio vulnificus* YJ016 and *Nostoc* sp. PCC 7120 are fusion proteins, having a C-terminal Cas1 domain but also a reverse transcriptase domain similar to that found in group II introns. This may represent one mechanism used for mobilization in a subset of CRISPR loci.

Clusters of *cas* genes and their associated repeats must maintain themselves in prokaryotic populations by reproducing and mobilizing themselves as fast as they are degraded. We see numbers of degenerate CRISPR/*cas* systems as well as profound differences in *cas* gene content between closely related species or strains. This is significant, because it implies that the process of replenishing genomes with intact CRISPR loci is frequent. We are inclined to believe that CRISPR/*cas* loci may, under certain circumstances, confer selective advantages to their host cells and, in these cases, stabilize the loci against degradation. We have yet to observe a single instance of a duplicated *cas* gene cluster on the chromosome(s) of any species. This is in contrast to selfish genetic elements such as transposons, which persist in a given lineage largely through redundancy.

Plasticity with respect to the number of repeat copies, as well as the extensive differences in the spacers between repeats, is observed in CRISPR loci [2,12,15,23]. The finding that spacer sequences derive from foreign DNA, such as phage and transposons, suggests a defensive capacity for at least some instances of CRISPR system [12,13], but roles in replicon partitioning in the Archaea [1] and regulation of fruiting body development in *M. xanthus* [19,22] are also suggested. Correlation of repeat expression with CRISPR subtype is in order. Apern subtype repeats are expressed and processed in *A. fulgidus* and *S. solfataricus* [11]. Also expressed, in addition to their neighboring *cas* genes, are the Nmeni repeats of *Streptococcus agalactiae* (H. Tettelin and J. Dunning Hotopp, personal communication), the Mtube repeats of *Staphylococcus epidermidis* (S. Gill, personal communication), and the Hmari/Mtube/RAMP module region repeats of *T. maritima* (data not shown). Five separate markers from the Ecoli-type CRISPR array of *G. sulfurreducens* were up-regulated 2- to 3-fold when cells were grown with Fe(III) versus fumarate as electron acceptor [24].

We have characterized multiple distinct subtypes of CRISPR/*cas* loci and demonstrated profound differences in CRISPR system content between closely related strains and species. Beneficial roles may include defense of the host against foreign DNA [12,13] and regulation of the fruiting body development cycle by the DevR and DevS *cas* genes in the special case of *M. xanthus* [19,22]. These findings support an emerging model of CRISPR/*cas* systems. They appear to be portable adaptation modules for their host genomes. They are sufficiently unstable that degenerate forms are often seen and sufficiently mobile that multiple instances of LGT are apparent. Their repeat arrays consistently are among the most rapidly evolving loci seen in strain comparison studies, such that they are the basis of “spoligotyping” [23,25,26]. Both *cas* gene and repeat expression can be differentially regulated. They can be co-opted by their hosts for new regulatory systems, as seen for a pathway unique to *Myxococcus* in the interaction between the non-Cas protein FruA and Cas protein DevR. The adaptations they enable may be supplanted later by the evolution of more stable regulatory systems, but in the meantime they may be superbly useful in rapid adaptation, such as in the invasion of a new biological niche.

## Materials and Methods

### Identification of CRISPRs.

CRISPRs were identified by three methods. Arrays of exact or near-exact repeats were readily detected by REPfind, a part of the REPuter package [27–29]. Sample sequences from known arrays were used to identify additional, smaller repeat clusters by BLASTN (http://blast.wustl.edu). Finally, regions suspected to have few and/or poorly conserved (degenerate) repeats, including regions near otherwise unexplained *cas* gene clusters, were examined manually with the dot-matrix homology visualization tool dotter [30].

### Definition of CRISPR-associated (Cas) protein families.

Cas protein families were identified from the construction of specific HMMs and subsequently deposited in the TIGRFAMs database (http://www.tigr.org/TIGRFAMs) [31]. The construction of HMMs involves refining multiple sequence alignments (also known as seed alignments), building HMMs from these alignments, exhaustively searching protein sequence databases, and selecting cutoff scores above which are found only true positives and below which no false negatives are detected. HMMs for Cas1–Cas4 were recognized among families previously designated “conserved hypothetical protein,” or were constructed for the first time, according to descriptions of these families by Jansen and colleagues [3]. All proteins encoded between or near identifiable *cas* genes were searched against a series of in-house databases of all available protein sequences and of prokaryotic genome sequences. Those that showed a pattern of matching numbers of similarly positioned proteins were investigated further as candidate new Cas protein families. For many of these families the process is iterative. Significant matches to the current model for the emerging family are found only near CRISPRs (see below) and/or previously identified *cas* genes. These new sequences are added to the family and realigned, and the revised HMM may then identify additional sequences. More distantly related sequences were distinguished from spurious matches by their contiguity to *cas* and CRISPR loci in other genomes, by the quality of the revised multiple sequence alignments, and by the improved search sensitivity of the HMM that resulted from adding these sequences to the seed alignment. Completed models were classified as new Cas protein families only if they did not overlap with other Cas HMMs, did not identify a sequence in a species that lacks CRISPRs, and if members of the family were found in at least four different species from at least three different lineages. Furthermore, members of these models had to be encoded adjacent or near to a set of CRISPRs and other *cas* genes. Iteration was halted rather than allow separate families to coalesce into one if the separate families showed substantially different domain architecture with only local sequence similarity, or if the separate families described separate genes recurringly found near one another in different genomes. The construction of Cas protein HMMs continued in this way until the boundaries of the loci were reached, where additional genes had identifiable non-CRISPR-associated functions, and/or their homologs in other genomes were no longer CRISPR-associated. All designated Cas family HMMs were searched routinely against comprehensive protein databases, which include eukaryotic sequences and CRISPR-negative prokaryotic genomes, to reconfirm specific association with repeats.

### CRISPR genome properties.

The presence or absence of CRISPR/Cas systems (with both repeats and sets of Cas proteins that include Cas1) in general and of eight different CRISPR/Cas subtypes (Ypest, Ecoli, Nmeni, Apern, Dvulg, Tneap, Hmari, Mtube, and the RAMP module) are determined by evidence-based rules implemented in the Genome Properties system [32]. Genome Properties is a database system (http://www.tigr.org/Genome_Properties) that can collect both manually curated and automated rule-based assertions of the presence or absence of complex biological systems and their components. States of YES and NO were imported from the work of Jansen and colleagues [3] and corrected in one case (*T. acidophilum* to YES). The YES state is set for subsequent prokaryotic genomes if Cas1 is detected; repeats are examined subsequent to assignment of the state. The state “none found” is converted to “NO” only if repeats prove absent. Rules for the individual CRISPR/Cas subtypes are based on protein family assignments made by the sets of subtype-specific HMMs listed in Table 1 and on proximity to the specified core Cas proteins for each type listed in Table 3.

## Abbreviations

Cas: CRISPR-associated
COGs: clusters of orthologous groups
CRISPR: clustered regularly interspaced short palindromic repeat
HMM: hidden Markov model
LGT: lateral gene transfer
LR: long repeat
RAMP: repair-associated mysterious protein
SR: short repeat
